# The stress-induced SCP/HLIP family of small light-harvesting-like proteins (ScpABCDE) protects Photosystem II from photoinhibitory damages in the cyanobacterium *Synechocystis* sp. PCC 6803

**DOI:** 10.1007/s11120-017-0426-3

**Published:** 2017-08-09

**Authors:** Tania Tibiletti, Ateeq Ur Rehman, Imre Vass, Christiane Funk

**Affiliations:** 1 Department of Chemistry, Umeå University, 90187 Umeå, Sweden; 2 Institute of Plant Biology, Biological Research Center, Szeged, Hungary; 3 Present Address: SC Synchrotron SOLEIL, AILES beamline, L’Orme des Merisiers Saint-Aubin- BP 48, 91192 Gif-sur-Yvette, France

**Keywords:** Small CAB-like proteins (SCPs), Photosystem II photoinhibition, Singlet oxygen, Salt stress, Terminal oxidases

## Abstract

Small CAB-like proteins (SCPs) are single-helix light-harvesting-like proteins found in all organisms performing oxygenic photosynthesis. We investigated the effect of growth in moderate salt stress on these stress-induced proteins in the cyanobacterium *Synechocystis* sp. PCC 6803 depleted of Photosystem I (PSI), which expresses SCPs constitutively, and compared these cells with a PSI-less/ScpABCDE^−^ mutant. SCPs, by stabilizing chlorophyll-binding proteins and Photosystem II (PSII) assembly, protect PSII from photoinhibitory damages, and in their absence electrons accumulate and will lead to ROS formation. The presence of 0.2 M NaCl in the growth medium increased the respiratory activity and other PSII electron sinks in the PSI-less/ScpABCDE^−^ strain. We postulate that this salt-induced effect consumes the excess of PSII-generated electrons, reduces the pressure of the electron transport chain, and thereby prevents ^1^O_2_ production.

**Electronic supplementary material:**

The online version of this article (doi:10.1007/s11120-017-0426-3) contains supplementary material, which is available to authorized users.

## Introduction

In oxygenic photosynthesis, light energy is used to drive two specialized protein complexes called Photosystem I (PSI) and Photosystem II (PSII), which are embedded in a special membrane system, the thylakoid membrane. Pigmented antenna proteins collect and transfer the sunlight to the photosystem reaction centers, where charge separation takes place. Concomitant with the electron transfer via the photosystems, protons are pumped through membrane-inserted complexes and create a chemo-osmotic gradient, required for the synthesis of ATP. ATP and NADPH are finally used in the biochemical reduction of carbon, nitrogen, and sulfur. In cyanobacteria, thylakoid membranes are not just the site of photosynthesis, but also of respiration (Vermaas 2001). Respiration and photosynthesis share many components including plastoquinones (PQ), the cytochrome *b*_6_ *f *(Cyt *b*_6_ *f*), and the two soluble redox carriers, plastocyanin (PC) and Cyt *c*_6_. In the model cyanobacterium *Synechocystis* sp. PCC 6803 (hereafter *Synechocystis*), PQ can be reduced either by PSII or by the NADP(H) dehydrogenase-like complex type I (NDH-1), the succinate dehydrogenase (SDH), and different NDH-2s (Cooley and Vermaas 2001). Electrons are transferred directly from plastoquinol (PQH_2_) to the *bd* quinol oxidases (Cyd; Berry et al. 2002) or via Cyt *b*_6_ *f* to PSI or an aa3-type cytochrome c oxidase (Cox) complex (Howitt and Vermaas 1998; Ermakova et al. 2016). In addition, there is the alternative respiratory terminal oxidase (ARTO). The NDH-1, SDH, and NDH-2 complexes and Cyd are ubiquitous in the thylakoid and the cytoplasmic membranes, while Cox and ARTO have been located only in the thylakoid or cytoplasmic membrane, respectively.

Although light is essential for photosynthesis, an excess of light energy can damage the photosynthetic apparatus in a process called photoinhibition, where PSII is the major site of damage (Aro et al. 1993). Excess of energy that cannot be used to drive photosynthesis enhances the production of reactive oxygen species (ROS) and induces photooxidative damages. A ROS marker for PSII damage is singlet oxygen (^1^O_2_) that is produced by the reaction of excited chlorophyll in its triplet state (^3^Chl*) with O_2_. When absorbed light cannot be fully utilized for electron transfer reactions, the probability of ^1^O_2_ formation increases. To dissipate excess excitation, photosynthetic organisms have developed different mechanisms. The fastest mechanisms are generally known as nonphotochemical quenching (NPQ; Niyogi 1999; Kirilovsky and Kerfeld 2012). They occur at the antenna level and they function by converting the excess light energy into heat through carotenoid molecules. In *Synechocystis*, an orange carotenoid protein (OCP) has been found to be the main component responsible for NPQ. OCP binds a single xanthophyll, 3′-hydroxyechinenone, whose absorption of blue-green light induces a conformational change of the protein that converts from an inactive form (OCP^O^) to an active red form (OCP^r^). OCP^r^ then binds to the phycobilisomes, the antenna of most species of cyanobacteria, quenching excess energy (for review see Kirilovsky and Kerfeld 2012).

An alternative to photoprotection via NPQ is to increase the electron sink capacity, i.e., enhance the capacity to consume photochemically generated electrons through the reduction of CO_2_ and O_2_. Several alternative electron pathways have been identified in cyanobacteria, higher plants, and algae involved in the dissipation of excess of light (McDonald et al. 2011; Chaux et al. 2015; Peltier et al. 2010; Roach and Krieger-Liszkay 2014).

*Synechocystis* contains five small CAB-like proteins (SCPs, named ScpA–E; Funk and Vermaas 1999, or also called high-light-induced proteins, HLIPs; Dolganov et al. 1995), which are induced during general stress, including light stress (He et al. 2001). SCPs consist of a single membrane-spanning helix, which has high homology to the first and third transmembrane regions of the higher plant light-harvesting complex. ScpA is the C-terminal extension of the ferrochelatase HemH, an enzyme involved in heme biosynthesis (Funk and Vermaas 1999; Sobotka et al. 2008, 2011; Storm et al. 2013); ScpB–E are proteins of around 6 kDa and have been found to be associated with PSII (Promnares et al. 2006; Yao et al. 2007; Kufryk et al. 2008; Shi et al. 2012). SCPs have been proposed to function in exciton dissipation (Havaux et al. 2003), to act as chlorophyll (Chl) carriers during assembly/repair of PSII (Knoppová et al. 2014; Hernandez-Prieto et al. 2011; Yao et al. 2012), or to regulate Chl biosynthesis (Xu et al. 2002, 2004).

While in wild-type *Synechocystis* SCPs only are expressed during stress, in a PSI-less background strain (Shen et al. 1993) they are expressed constitutively (Funk and Vermaas 1999). The PSI-less/ScpABCDE^−^ mutant appears chlorotic compared to the control PSI-less strain due to decreased PSII content, and glycogen accumulates in the cell (Hernandez-Prieto et al. 2011; Tibiletti et al. 2016). It has been proposed that the absence of SCPs decreases the stability of the Chl-binding proteins within PSII and leads to the formation of ROS (Hernandez-Prieto et al. 2011; Sinha et al. 2012).

Comparing the PSI-less/ScpABCDE^−^ mutant with the SCP-expressing PSI-less control strain, here we show that in the presence of 0.2 M sodium chloride in the growth medium the control phenotype is restored in the PSI-less/ScpABCDE^−^ mutant. We propose that SCPs protect PSII from photoinhibitory damages by decreasing the ^1^O_2_ production.

## Materials and methods

### Growth conditions, cell counting, and measurement of cell size

For each experiment, *Synechocystis* PSI-less (Shen et al. 1993) and PSI-less/ScpABCDE^−^ (Xu et al. 2004) mutants were first plated freshly from frozen stock cultures and then cultivated in flasks with BG-11 medium (Rippka et al. 1979) shaken at 100 rpm at 28 °C and low light intensity (4–5 μmol photons m^−2^ s^−1^). Different frozen stocks were used for the biological replicates to avoid effects of secondary mutations. The liquid growth medium was supplemented with 10 mM glucose and 10 mM TES–NaOH, pH 8.0. For growing on plates, solid BG-11 medium was supplemented with 10 mM glucose, 10 mM TES–NaOH, pH 8.2, and 20 mM Na-thiosulfate.

Cell precultures were inoculated in liquid BG-11 medium supplemented with 10 mM glucose in the presence or absence of 0.2 M NaCl and allowed to acclimate for 3 days (corresponding roughly to four generations). In exponential logarithmic growth, an inoculum of these precultures was then used to inoculate fresh medium (in the presence or absence of 0.2 M NaCl) at an optical density at 730nm (OD_730_) of 0.2 for cultures used in the experiments. The concentration of 0.2 M sodium chloride in the medium to induce salt stress was chosen based on the study by Howitt et al. (2001) determining the salt tolerance of different *Synechocystis* mutants. The influence of pH on the mutants was tested by adding 20 mM TES–NaOH to obtain pH 8, 20 mM of CAPS (pH 9), or 20 mM of MES–NaOH to obtain pH 6.5 to the medium. A final concentration 0.5 mM NaHCO_3_ was added when indicated in a buffered (pH 8) BG-11 medium supplemented with glucose. In this case, cells precultured in liquid BG-11 medium supplemented with 10 mM glucose were inoculated in media with different pH or in the presence of NaHCO_3_. Each experiment was performed with three biological replicates. To ensure that the culture was in exponential growth phase at the beginning of the experiment, the cells were counted using a Neubauer improved chamber. Growth of the cultures was also monitored by measuring the OD_730_ using a T90+ spectrophotometer (PG Instruments), OD_730_ 0.5–0.8 corresponding to a logarithmic growth phase. Cell size was measured using the Multisizer™ 4 Coulter Counter^R^ (Beckman Coulter).

### Pigment determination

Chl was extracted from total cells using 100% methanol and its concentration was determined from the absorbance at 665 nm using the extinction coefficient for Chl *a* in methanol of 71.43 mM^−1^ cm^−1^ taken by Porra (2002). The carotenoid/Chl (Car/Chl *a*) ratio was calculated from the pigment absorbance at 664 nm (for Chl *a*) and 474 nm (for carotenoids).

### Low-temperature fluorescence

Low-temperature fluorescence spectra were recorded using a FluoroMax-2 fluorometer (Spex spectrofluorometer system, Jobin Yvon, Longjumeau, France). Cells after growth in the presence or absence of NaCl were concentrated to 1 mg Chl *a*/mL and were frozen without glycerol to avoid functional uncoupling of the phycobilisomes from thylakoid components. The obtained spectra were normalized to their maximum values (Shen and Vermaas 1994).

### SDS-PAGE and immunoblot analysis

An equal number of cells were pelleted. After Chl extraction, the proteins were resuspended in SDS-loading buffer (245 mM Tris–HCl, pH 8.2, 0.5 mM ethylenediaminetetraacetic acid (EDTA), 2% lithium dodecyl sulfate (LDS), 10% glycerol, and 50 mM dithiothreitol (DTT)) and heated at 65 °C for 15 min. After the removal of unsolubilized proteins by centrifugation (20,000 g for 10 min), the sample was loaded onto a sodium dodecyl sulfate (SDS)-acrylamide gel with 6 M urea. Immunoblotting using antibodies against the PSII proteins D1, CP47, and PsbH, as well as against ScpC/ScpD and ScpE, was performed as described in Hernandez-Prieto et al. (2011).

### Oxygen evolution and respiration measurements

In cultures with an OD_730_ of 0.6, the electron transport activity of PSII and the dark respiration were measured at 30 °C with a Clark-type oxygen electrode (Hansatech Instrument). The oxygen-evolving activity of PSII was measured in BG-11 medium supplemented with 10 mM glucose in the presence of 600 μM 2,6-dichloro-*p*-benzoquinone (DCBQ) and 1.2 mM potassium ferricyanide (FeCN) under saturating light intensity (2000 μmol photons m^−2^ s^−1^).

For respiration measurements, an early logarithmic culture was harvested and resuspended in fresh BG-11 containing 10 mM glucose. The cell density was adjusted to a chlorophyll concentration of 10 μM. 1 mL of this concentrated culture was assayed for the capacity to consume O_2_ in the dark. 15 mM potassium cyanide (KCN) was added to the culture to inhibit the respiratory activity and to obtain the baseline, following Wang et al. (2012).

### Histidine-mediated oxygen uptake measurement

For quantification of singlet oxygen production, histidine-mediated oxygen uptake measurements were applied, which are based on the oxidation of histidine by ^1^O_2_. The subsequent removal of dissolved oxygen in aqueous media was detected by a standard oxygen electrode (Rehman et al. 2013). The rate of singlet oxygen-induced oxygen uptake was measured in the presence of 5 mM His at 2300 µmol photons m^−2^ s^−1^ light intensity using a Hansatech DW2 O_2_ electrode at a Chl concentration of 2 µg mL^−1^ in the absence of artificial electron acceptors as described earlier (Rehman et al. 2013). Before the O_2_ uptake measurements, *Synechocystis* cells were centrifuged and resuspended in fresh BG-11 medium.

### Photoinhibition measurements

Photoinhibition measurements were performed as described in Hernandez-Prieto et al. (2011) and Rehman et al. (2013). High-light illumination experiments were performed in open, square glass containers, in which the cell suspension formed a 14-mm-high layer, with continuous stirring at 25 °C. An array of 50 W halogen lamps with adjustable light intensities provided the homogenous white light illumination of 200 µmol photons m^−2^ s^−1^. PSII activity was assayed by the initial amplitude of the flash-induced Chl fluorescence signal.

### Attenuated total reflection Fourier transform infrared (ATR FT-IR) spectroscopy

A volume of 25 mL of cells was harvested by centrifugation at 4000 rpm and washed twice with water. The cell pellet was frozen in liquid nitrogen, freeze-dried, and stored until analysis. FT-IR measurements were carried out with a Tensor 27 Spectrophotometer (Bruker). A tip of a spatula of freeze-dried cells was resuspended in water and applied directly on a crystal surface. After drying under air flux, 500 scans over 7 min were recorded for each sample, within the range of 3500–800 cm^−1^ at a resolution of 8 cm^−1^. A background spectrum was recorded before each sample measurement and automatically subtracted from the sample spectrum. Spectra were cut to 1850–800 cm^−1^, baseline-corrected, and normalized to the total peak area. Spectra were analyzed using OPUS v. 5.5 software.

### Statistics

Data are reported as the mean ± standard deviation of three or more biological replicates. Statistical significance of differences among the means was determined by an unpaired two-tailed *t* test when only two groups were compared, or by one-way analysis of variance (ANOVA) and Tukey’s post hoc test when more than two groups were compared. GraphPad Prism 4.03 software (GraphPad Software, San Diego, CA, USA) was used, with the level of significance set at 95%. *P* values were classified as follows: <0.001 extremely significant***, 0.001–0.01 very significant**, and 0.01–0.05 significant*.

## Results

### **NaCl induces pigment biosynthesis and PSII assembly in the** PSI-less/ScpABCDE^−^**strain**

**Fig. 1:**
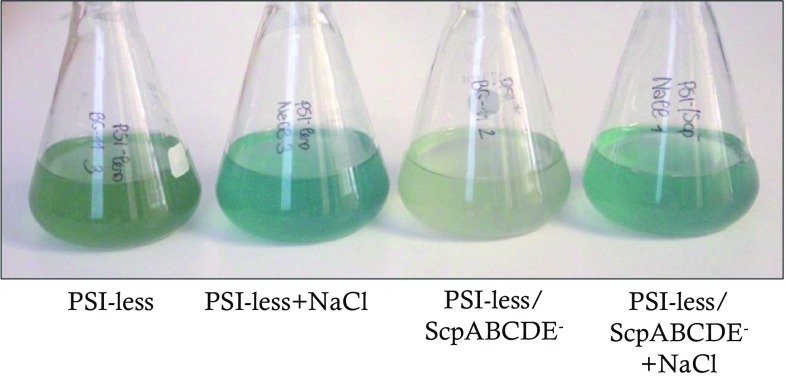
Color appearance of the PSI-less and PSI-less/ScpABCDE^−^ mutants grown in the presence or absence of 0.2 M NaCl. Cell cultures of PSI-less and PSI-less/ScpABCDE^−^ strains were in BG-11 supplemented with glucose in the presence or absence of 0.2 M NaCl as described in Material and Methods. The photo was taken after dilution at OD_730_ 0.4–0.5. Representative cultures of three biological replicates are shown

The SCP-expressing PSI-less mutant and the PSI-less/ScpABCDE^−^ strain were cultured in BG-11 medium supplemented with 10 mM glucose in the presence or absence of 0.2 M NaCl. Under control growth conditions (4 µmol photons m^−2^ s^−2^ in continuous light at 30 °C, absence of NaCl), the PSI-less/ScpABCDE^−^ strain appeared chlorotic compared to the PSI-less control strain (Fig. 1). In the presence of 0.2 M NaCl, however, the pigmentation was affected in both strains with higher impact on the PSI-less/ScpABCDE^−^ mutant, as could be observed by eye (Fig. 1) and by measuring the pigment absorption (Table 1). On cell basis, the amount of Chl *a* increased by 4.6 times in the PSI-less/ScpABCDE^−^ strain (*p* < 0.001), while the amount of Chl *a* in the PSI-less control doubled (*p* < 0.05, Table 1). The cell division time of the PSI-less/ScpABCDE^−^ strain was not significantly altered in the presence of NaCl (*p* > 0.05, Table 1), while doubling of the PSI-less control strain decreased 1.6 times (*p* < 0.05, Table 1).

**Table 1:** Comparison of the PSI-less/ScpABCDE^−^ mutant and the PSI-less control strain grown in the presence or absence of 0.2 M NaCl

	Division time (hours)	Cell diameter (μm)	nmol Chl *a* mL^−1^ OD_730_	fmol Chl *a* cell^−1^	Car/Chl *a*	O_2_ evolution fmol O_2_ h^−1^ cell^−1^	Respiration fmol O_2_ consumption h^−1^ cell^−1^
PSI-less	27.38 (± 1.33)	1.67 (± 0.03)	0.63 (± 0.01)	3.92 (± 0.05)	2.11 (± 0.01)	13.99 (± 1.57)	0.82 (± 0.06)
PSI-less+NaCl	43.4* (± 4.1)	1.7 (± 0.01)	0.87*** (± 0.02)	8.22* (± 0.27)	1.51*** (± 0.02)	33.52** (± 7.28)	1.08 (± 0.15)
PSI-less/ScpABCDE^−^	37.59 (± 3.74)	2.13*** (± 0.03)	0.23*** (± 0.01)	3.68 (± 0.03)	3.36*** (± 0.16)	8.81 (± 2.03)	1.63*** (± 0.10)
PSI-less/ScpABCDE^−^+NaCl	44.6 (± 11.67)	2.23** (± 0.04)	0.67*** (± 0.04)	16.85*** (± 3.61)	1.78*** (± 0.1)	47.72*** (± 4.46)	3.04*** (± 0.18)

Both strains were cultivated in BG-11 standard medium supplemented with 10 mM glucose. Standard deviations with *n* ≥ 3 are given in brackets

*P* values are classified as: *0.01 to 0.05 significant; **0.001 to 0.01 very significant; ***< 0.001 extremely significant

**Fig. 2:**
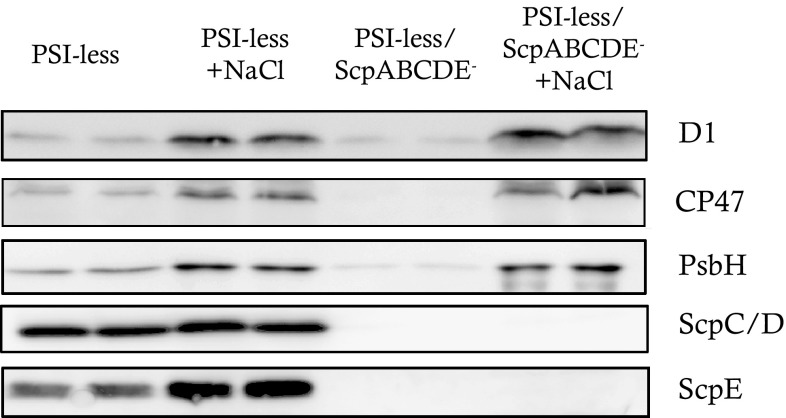
Immunodetection of the PSII proteins D1, CP47, and PsbH, as well as of ScpC/D and ScpE, in the PSI-less and the PSI-less/ScpABCDE^−^ strains, when grown in the presence or absence of 0.2 M NaCl. Proteins extracted from the same number of cells were loaded in each lane. A representative immunoblot of three biological replicates is shown

Both strains, the PSI-less/ScpABCDE^−^ and the PSI-less control, contained more Chl when grown in the presence of NaCl. Free Chl is potentially damaging the cell and therefore scavengers like a carotenoid molecule have to be in its proximity. To analyze if the NaCl-induced Chl molecules were used to assemble additional PSII complexes, oxygen evolution measurements were performed using a Clark electrode (Table 1). Indeed, cells grown in BG-11 supplemented with NaCl evolved more oxygen; while more than five times O_2_ h^−1^ was produced per cell by the PSI-less/ScpABCDE^−^ strain grown in the presence of NaCl (*p* < 0.001, Table 1), oxygen evolution of the PSI-less culture only slightly increased. Immunoblotting experiments were performed to confirm the biosynthesis of more PSII complexes per cell (Fig. 2). Proteins were extracted from the same number of cells of the PSI-less/ScpABCDE^−^ and the PSI-less control strain, which had been grown in the presence or absence of NaCl for 4 days. After SDS-PAGE, several proteins were immunostained with specific antibodies: the PSII reaction center protein D1, the PSII core antenna protein CP47, and the low-molecular weight PSII protein PsbH. Additionally, antibodies recognizing ScpC/ScpD or ScpE were used. In untreated cells, the PSI-less/ScpABCDE^−^ strain contained very few PSII centers (judged by the presence of D1, CP47, and PsbH) per cell (Fig. 2), confirming earlier results (Hernandez-Prieto et al. 2011). In the PSI-less control, more PSII proteins were detected, while the SCPs were strongly expressed (Funk and Vermaas 1999). Presence of NaCl further stimulated SCP expression in the control strain with ScpE accumulating stronger per cell than ScpC/D. Interestingly, while the presence of NaCl induced an increasing amount of PSII proteins in both strains, its impact in the PSI-less/ScpABCDE^−^ mutant per cell was much stronger compared to the PSI-less control.

**Fig. 3:**
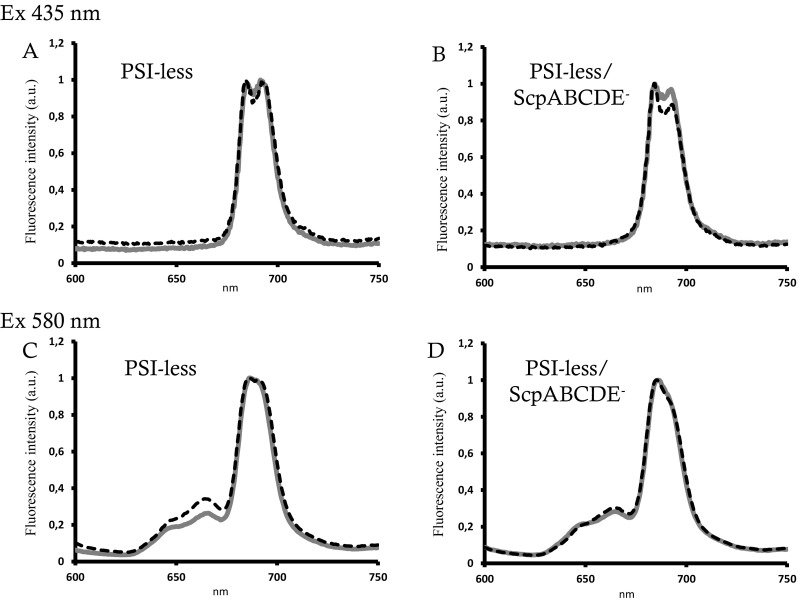
77 K fluorescence spectra of the PSI-less (*left*) and the PSI-less/ScpABCDE^−^ mutant (*right*). Spectra were recorded from 600 to 750 nm after growth in the absence (*black line*) or presence (*gray line*) of 0.2 M NaCl and normalized to the 685 nm peak. **a** Excitation of chlorophyll at 435 nm. **b** Excitation of phycobilisomes at 580 nm

Low-temperature (77 K) fluorescence emission spectra offer good resolution of the fluorescence produced by PSII components. Additionally, selection of the emission wavelength allows relative quantification of the ratio between antenna and photosystem pigments. Upon excitation at 435 nm, which excites mainly Chl *a*, two peaks with maximum at 685 and 695 nm were observed. The peak at 685 nm reflects all Chls associated with PSII (including Chl from CP43, D2, D1, and CP47), except for one low-energy Chl *a* 627 that appears to be associated with His114 of CP47. This red low-energy Chl is the main contributor to the 695 nm emission maximum (Vermaas et al. 1986; Shen and Vermaas 1994). In the PSI-less control strain, fluorescence spectra were similar, independent of growth in the presence or absence of NaCl (Fig. 3a). However, when Chl (435 nm) was excited in the PSI-less/ScpABCDE^−^ strain grown in the presence of 0.2 M NaCl, the fluorescence maximum at 695 nm increased compared to the control cells grown in the absence of NaCl (Fig. 3b).

Excitation of the phycobilisomes at 580 nm showed a decrease of phycobilisome fluorescence in the PSI-less control after growth in the presence of salt indicating an improved energy transfer between phycobilisomes and PSII or increased OCP-mediated NPQ (Fig. 3c). On the contrary, no difference in fluorescence upon growth in the presence of NaCl was observed in the PSI-less/ScpABCDE^−^ strain (Fig. 3d). The higher 695 nm emission after 435 nm excitation observed in the PSI-less/ScpABCDE^−^ strain upon growth in the presence of salt could therefore originate from a disturbed energy transfer between CP43 and CP47 and/or from a structural rearrangement around the PSII antenna CP47 (D’Haene et al. 2015).

### Enhanced cell pigmentation is independent of pH/osmolarity

To understand the effect of NaCl on the phenotype of the PSI-less/ScpABCDE^−^ mutant and to distinguish the salt effect from a possible pH/osmolarity effect, the mutant strain and its PSI-less control were grown in BG-11 medium buffered to pH 8 (standard), pH 6.5, or pH 9, or NaHCO_3_ was added at a final concentration of 0.5 mM (Supplementary Fig. 1 and Supplementary Table 1). While at pH 9 the amount of Chl *a* per cell increased in both strains (accompanied with an increased doubling time) (Supplementary Table 1), at pH 6.5 no significant differences to the standard pH of 8 were observed in the PSI-less strain. Notably, at pH 6.5 the PSI-less/ScpABCDE^−^ strain was not viable, and it died within 4 days after inoculation. The growth rate in the presence of 0.5 mM HCO_3_^−^ was diminished in both strains (by a factor of 1.5 for the PSI-less and 2 for the PSI-less/ScpABCDE^−^ strain) compared to standard conditions (*p* < 0.001, Supplementary Fig. 1, Supplementary Table 1), and the amount of chlorophyll per cell was slightly, but not significantly, lower in both strains when grown in the presence of HCO_3_^−^ compared to growth at pH 8 (*p* > 0.05, Supplementary Table 1). HCO_3_^−^ is transported into the cyanobacterial cell via a symporter system powered by Na^+^; increased HCO_3_^−^ concentration in the medium could therefore induce effects similar to increased NaCl concentration. Based on these data, we conclude that NaCl directly affects the cell growth, and a secondary effect based on pH osmolarity can be excluded.

### **NaCl increases the respiration rate in the** PSI-less/ScpABCDE^−^**mutant**

Growth in the presence of NaCl has been shown to increase the respiratory activity in *Synechocystis* wild-type cells (Jeanjean et al. 1990, 1993). To analyze if a similar effect could be observed in the two PSI-less mutants used in this study, the respiration rates of the PSI-less control and the PSI-less/ScpABCDE^−^ strain were measured in the presence or absence of NaCl (Table 1). In standard BG-11 medium, the respiration rate of the PSI-less/ScpABCDE^−^ mutant was twice as high as in the PSI-less control. Growth in the presence of 0.2 M NaCl led to an additional doubling of its respiration rate (*p* < 0.001, Table 1), while respiration of the PSI-less control only slightly increased. NaCl therefore strongly affects the respiration rate of the PSI-less/ScpABCDE^−^ mutant and concomitantly induces a higher flux of electrons through the respiration chain.

### **NaCl prevents the production of singlet oxygen in the** PSI-less/ScpABCDE^−^**mutant**

**Fig. 4:**
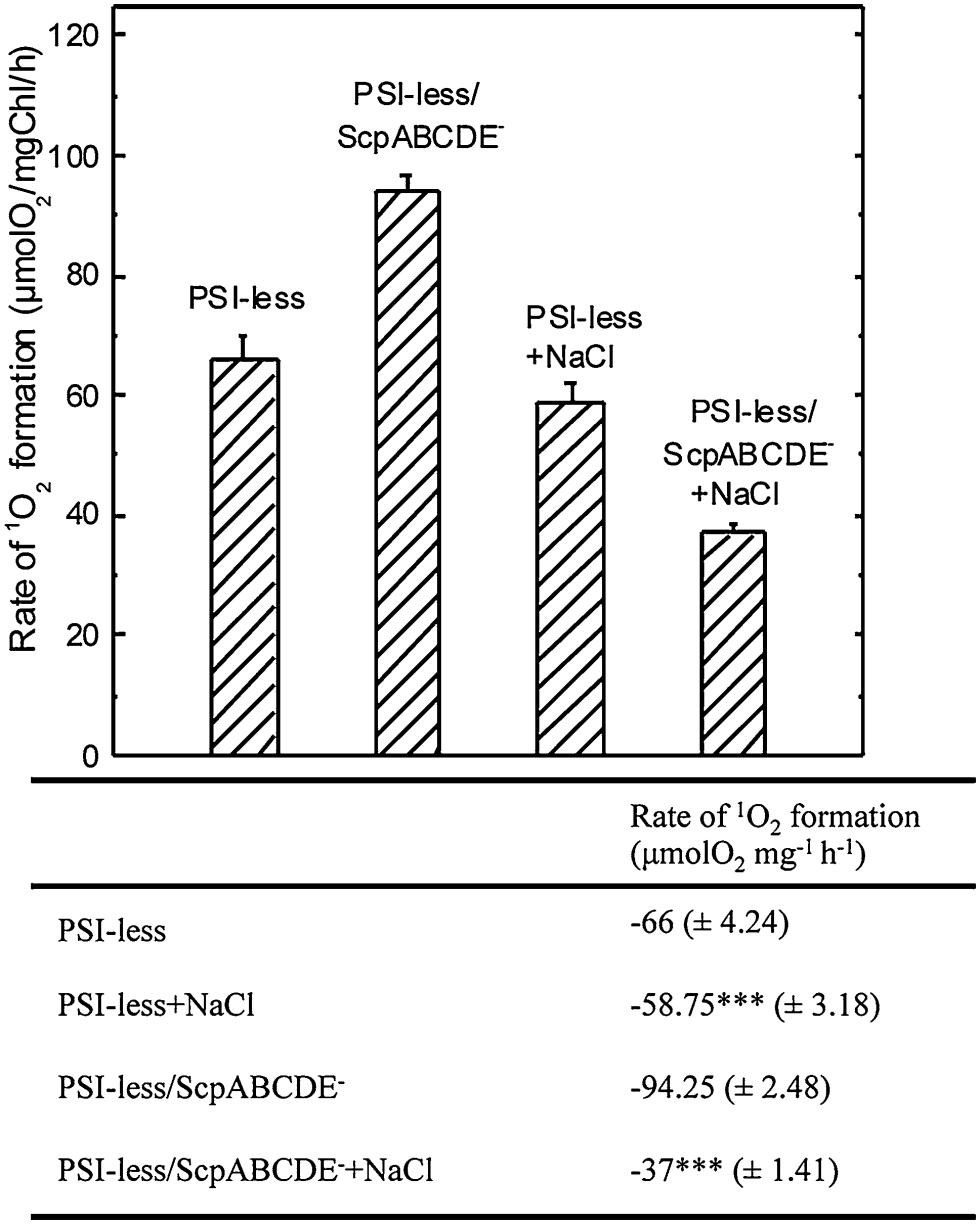
Measurement of ^1^O_2_ production expressed as the inverse rate of His-mediated oxygen uptake in the PSI-less and the PSI-less/ScpABCDE^−^ strains grown in the presence or absence of NaCl. The measurements were performed in the presence of 5 mM His at 2300 μmol photon m^−2^ s^−1^ light intensity. The results are a mean (± SD) of three independent experiments. *P* values are classified as: *0.01 to 0.05 significant; **0.001 to 0.01 very significant; ***< 0.001 extremely significant

^1^O_2_ is highly damaging in cells, and the SCPs have been suggested to prevent ROS damage (Sinha et al. 2012). Based on our result that the biosynthesis of chlorophyll and the assembly of PSII complexes are enhanced in the presence of NaCl in the PSI-less/ScpABCDE^−^ strain, the effect of NaCl on ^1^O_2_ was measured in intact mutant cells using chemical trapping of histidine (Rehman et al. 2013). As expected, an increased rate of ^1^O_2_ production was observed in the PSI-less/ScpABCDE^−^ mutant, 1.4 times higher than in the PSI-less strain, as calculated from the rate of O_2_ uptake due to oxidation of histidine by ^1^O_2_ (*p* < 0.001, Fig. 4). In the presence of 0.2 M NaCl, the production rate of ^1^O_2_ slightly (non-significantly) decreased in the PSI-less control (*p* > 0.05, Fig. 4). However, upon growth in the presence of 0.2 M NaCl the rate of ^1^O_2_ production of the PSI-less/ScpABCDE^−^ strain was decreased by 2.6 times relative to the value obtained in standard BG-11 (*p* < 0.001, Fig. 4). In fact, in the presence of NaCl the ^1^O_2_ production rate of the PSI-less/ScpABCDE^−^ mutant was only half of the one in the PSI-less control.

### **NaCl leads to decreased carbon**/**nitrogen (C**/**N) ratio in the** PSI-less/ScpABCDE^−^**strain**

Attenuated total reflectance Fourier Transform Infrared (ATR FT-IR) spectroscopy is a rapid and inexpensive method to ensure the cell fitness by measuring the C/N ratio. Using this method, we had previously observed a strong increase of the C/N ratio in the PSI-less/ScpABCDE^−^ strain compared to the PSI-less control, being a symptom of oxidative damage and ultimately reduced fitness of the cells (Tibiletti et al. 2016). These findings could be confirmed by measuring ATR FT-IR on both mutants upon growth in BG-11 medium supplemented with 10 mM glucose (Table 2). However, after growth in BG-11 supplemented with glucose and 0.2 M NaCl, the C/N ratio in the PSI-less/ScpABCDE^−^ strain significantly decreased (*p* < 0.001) by more than half, becoming comparable to the PSI-less strain cultivated in standard conditions (Table 2), while NaCl in the PSI-less control strain only induced a decrease of 30%. Growth in the presence of NaCl therefore seems to influence not only the electron transport chain, but also the entire cell metabolism.

**Table 2:** C/N ratios of the PSI-less control and the PSI-less/ScpABCDE^−^ mutant in the presence or absence of NaCl calculated after ATR FT-IR

	C/N
PSI-less	0.63 (± 0.12)
PSI-less+NaCl	0.44* (± 0.1)
PSI-less/ScpABCDE^−^	1.38*** (± 0.06)
PSI-less/ScpABCDE^−^+NaCl	0.75*** (± 0.05)

Standard deviations with *n* ≥ 3 are given in brackets

*P* values are classified as: *0.01 to 0.05 significant; **0.001 to 0.01 very significant; ***< 0.001 extremely significant

### PSII photoinhibition

**Fig. 5:**
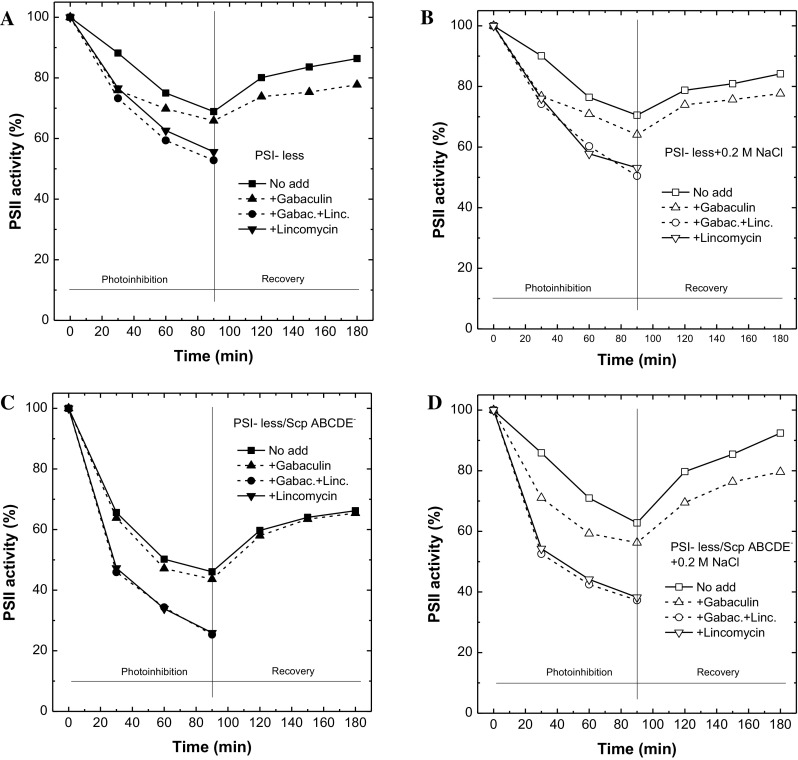
Measurements of photodamage and PSII repair of the PSI-less (*upper panels*) and PSI-less/ScpABCDE^−^ (*lower panels*) mutants grown in the presence (*right, open symbols*) or absence (*left, closed symbols*) of 0.2 M NaCl. Photoinhibitory treatment was performed in the presence (*down triangle symbol, solid line and circle symbol, dotted lines*) or absence (*square symbol, solid line and upper triangle symbol, dotted lines*) of 300 μg/mL lincomycin, as well as in the presence (*dotted lines*) or absence (*solid lines*) of 5 μM gabaculine

We monitored PSII photoinhibition in the PSI-less and PSI-less/ScpABCDE^−^ strains grown in standard BG-11 or in BG-11 supplemented with NaCl (Fig. 5). In the PSI-less control strain, PSII activity decreased to 70% after high light stress (90 min at 200 μmol photons m^−2^ s^−1^) and recovered to about 85% after 90 min in weak light, independent of the presence or absence of NaCl (Fig. 5a, b). Addition of gabaculine, an inhibitor of chlorophyll biosynthesis (Hill et al. 1985), and phycocyanin (dotted lines) led to further decrease of PSII activity during light stress (increased photoinhibition) and diminished recovery in weak light (to less than 80%; Fig. 5a, b, dotted lines). We conclude that the enhanced loss of PSII in the presence of gabaculine is related to a partial impairment of PSII repair, since the elimination of protein synthesis by the inhibitor lincomycin led to the same PSII activity loss (rate and extent) independent of the presence or absence of gabaculine (Fig. 5a, b, down triangle symbol, solid line and circle symbol, dotted lines).

The PSI-less/ScpABCDE^−^ mutant showed stronger photoinhibition compared to the PSI-less strain when grown in standard BG-11 supplemented with glucose (Fig. 5c), as observed earlier (Hernandez-Prieto et al. 2011). More than 50% of the PSII activity was lost, and weak light allowed recovery to 60% activity. In the presence of 0.2 M NaCl, however, only 30–40% of PSII became photoinhibited in the PSI-less/ScpABCDE^−^ mutant, and recovery was up to 90% (Fig. 5d). Interestingly, in the PSI-less/ScpABCDE^−^ strain grown in the absence of NaCl, addition of gabaculine had no effect, neither on its photoinhibition, nor on recovery (Fig. 5c, dotted line) (see also Hernandez-Prieto et al. 2011), while after growth in the presence of 0.2 M NaCl gabaculine induced a stronger photoinhibition (40% loss of PSII activity) and prevented full recovery (only up to around 80%). Even in this mutant addition of lincomycin induced the same rates independent of the presence or absence of gabaculine (Fig. 5c, d, open down triangle symbol, solid line and open circle symbol, dotted lines).

## Discussion

During salt stress, it is important for cyanobacteria like *Synechocystis* sp. PCC 6803 (Jeanjean et al. 1990, 1993; Peschek et al. 1994) or *Synechococcus* sp. PCC 6311 (Fry et al. 1986) to provide a proton gradient for Na^+^/H^+^ antiporters localized in the thylakoid or cytoplasmic membrane (Hagemann 2011). The ATP to drive these Na^+^/H^+^ antiporters is provided by oxidase activity and the respiratory electron transport (Hagemann 2011). Enhanced respiration in the presence of NaCl is also known as salt respiration (Paschinger 1977; Pils and Schmetterer 2001). In cyanobacteria, the respiratory electron transport shares common components with the photosynthetic electron transport (Scherer 1990; Schultze et al. 2009; Ermakova et al. 2016), and therefore respiration can function as a sink for PSII-generated electrons when needed (e.g., during excess of light). The efficiency of oxidases to function as electron sinks in *Synechocystis* (Vermaas et al. 1994; Berry et al. 2002; Howitt and Vermaas 1998; Nomura et al. 2006; Ermakova et al. 2016; Lea-Smith et al. 2013) and other photosynthetic organisms (Houille-Vernes et al. 2011; Laureau et al. 2013) has been shown earlier.

In *Synechocystis*, most Chl is bound to PSI, and mutants deficient of PSI contain only 20% of the wild-type chlorophyll amount (Shen et al. 1993). In PSI-less mutants, PSII is very vulnerable to photooxidation as PSII-generated electrons are not efficiently consumed by PSI (Sandström et al. 2002). As a consequence, PSI-less mutants are not able to grow at normal or high irradiances and express the stress-induced SCPs constitutively. Additional deletion of the five SCPs leads to further reduction in chlorophyll (Xu et al. 2004; Tibiletti et al. 2016). In this work, we show that the presence of 0.2 M NaCl restores the bleached phenotype of the PSI-less/ScpABCDE^−^ mutant strain. We propose that the presence of 0.2 M NaCl induces increased respiratory activity (Table 1) that consumes the excess of PSII-generated electrons, thereby reducing the pressure of the electron transport chain, lowering incidences of charge recombination, and preventing ^1^O_2_ production (Fig. 4). Lower ROS in turn reduces the risk for PSII photoinhibition (Fig. 5) and allows the accumulation of Chl *a* assembled into PSII (Table 1).

In the PSI-less/ScpABCDE^−^ mutant, the rate of oxygen evolution is higher than the respiration rate (Table 1); besides oxidases, other cell components have therefore to be involved in the consumption of PSII-generated electrons. Alternative electron sinks, such as the Flv2/Flv4 complex, which accepts electrons directly from PSII (Zhang et al. 2012), the hydrogenase complex, nitrate reductase, or carbon fixation might fulfill this function (Gutthann et al. 2007). The lower C/N ratio observed in PSI-less/ScpABCDE^−^ cells exposed to NaCl indicates the nitrate reductase and CO_2_ (possibly through a PQH_2_-oxidizing pathway, Wang et al. 2012) to serve as terminal acceptors. As respiration and photosynthesis are intertwined in cyanobacteria, we cannot exclude in PSI-less mutants the O_2_ uptake to vary during the light phase. In the absence of PSI, O_2_ uptake in light might be stimulated; in the cyanobacterium *Synechococcus elongatus*, growth in high light stress induced O_2_ uptake (Hoch et al. 1963).

Na^+^/H^+^ antiporters not only extrude Na^+^, but also are important to maintain the intracellular pH by transporting H^+^. However, recovery of the bleached phenotype in the PSI-less/ScpABCDE^−^ was pH independent (Supplementary Fig. 1 and Supplementary Table 1). Presence of HCO_3_^−^, which is mainly transported into the cyanobacterial cell via a symporter system powered by Na^+^ (Shibata et al. 2002), did not recover the phenotype either. These data suggest Na^+^ (or Cl^−^) ions and not H^+^ to be mainly responsible for the recovery of the PSI-less/ScpABCDE^−^ mutant. Interestingly, the PSI-less/ScpABCDE^−^ mutant died when grown at low pH. Already in our previous microarray data comparing the PSI-less/ScpABCDE^−^ to PSI-less control cells (Tibiletti et al. 2016), we observed a differential expression of several genes involved in ion homeostasis and inorganic carbon metabolism, indicating a low capacity to regulate pH/osmolarity of the mutant. For instance, *aqpZ* encoding an aquaporin, *sbta* encoding the Na^+^-dependent HCO_3_^−^ transporter, *nhaS3* encoding the only thylakoid localized Na^+^/H^+^ antiporter, and *pxcA* encoding an ATP-dependent proton extrusion system were found to be up-regulated in the PSI-less/ScpABCDE^−^ mutant compared to the PSI-less control. High H^+^ influx into the cell at low pH might block or saturate the light-induced proton extrusion system that is essential for growth, CO_2_ transport, and nitrate uptake at pH 6.5 (Sonoda et al. 1998; Katoh et al. 1996). In mixotrophic growth, inorganic carbon (Ci) transportation through the carbon concentration mechanisms (CCM) could be important to regulate the internal pH, pumping H^+^ out of the cell. This mechanism is most important at low pH when CO_2_ is the most abundant Ci species dissolved in water (Kirk 2011). The high C/N ratio observed in the PSI-less/ScpABCDE^−^ could block the full induction of CCM, leading to accumulation of H^+^ in the cell and finally to death. Unfortunately, we failed to measure the proton extrusion induced by NaCl and the pH difference across the membranes using the dye acridine yellow as described by Teuber and coworkers (Teuber et al. 2001).

At 77 K, the ratio of fluorescence at 695 and 685 nm (F695/F685) in the PSI-less/ScpABCDE^−^ mutant upon growth in the presence of NaCl was restored to approximately 1, similar to the PSI-less mutant, while during growth under standard conditions the 695 nm fluorescence peak was enlarged compared to the one at 685 nm (Fig. 3a, b). This might indicate a disturbed energy transfer between CP43 and CP47 and/or a structural rearrangement around the PSII antenna CP47 in PSI-less/ScpABCDE^−^. We propose that the lack of SCPs affects the structural organization of PSII in stressed conditions, possibly under the environment of the red-emitting Chl 627 of CP47 as suggested for a mutant deficient in PsbH (D’Haene et al. 2015). Association of SCPs to CP47 seems to be PsbH dependent and in the absence of PsbH and SCPs the environment of the Chl 627 of CP47 is modified. Thus, SCPs might effectively stabilize PSII complexes under stress conditions.

With the reduction of ^1^O_2_, a strong recovery from photoinhibition was observed in the PSI-less/ScpABCDE^−^ upon growth in NaCl (Fig. 5). The percentage of PSII recovering after photoinhibition in the PSI-less/ScpABCDE^−^ mutant upon growth with NaCl was similar to the PSI-less strain, both in the presence and absence of gabaculine. Gabaculine is an inhibitor of Chl biosynthesis that affects the synthesis of the 5-aminolevulinic acid and inhibits phycocyanin (Hill et al. 1985; Beale 1999). Hence, the cells grown in the presence of gabaculine can only repair or assemble new PSII with Chl molecules recycled from damaged PSII. Comparing the relative loss of PSII activity in the presence or absence of gabaculine, the shortage of Chl molecules affects strongly the photoinhibitory period (mostly during the first 20 min) than the recovery. Addition of gabaculine had no additional effect on photoinhibition and recovery indicating that the *novo* assembly and repair of PSII in the PSI-less/ScpABCDE^−^ mutant grown in the absence of NaCl relies only on Chl recycling. Based on the data presented here and in Hernandez-Prieto et al. (2011), we calculated 5–15% of PSII repair to depend on newly synthesized Chl molecules during photoinhibition and 5% during recovery in PSI-less and PSI-less/ScpABCDE^−^ upon growth with NaCl. This calculation was performed considering the PSII activity in the presence or absence of gabaculine at 90 and 180 min.

**Fig. 6:**
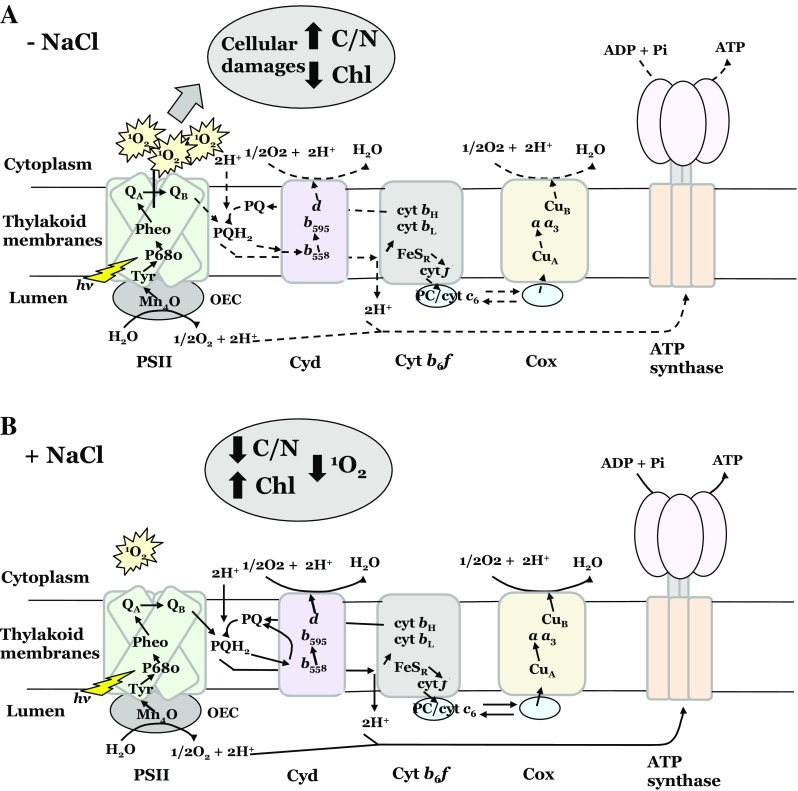
Schematic diagram of the thylakoid membrane-localized photosynthetic and respiratory electron transport chains in PSI-less/ScpABCDE^−^ in the absence (**a**) or presence (**b**) of NaCl. *Lines* indicate the electron transport, *filled lines* indicate sustained electron transfer, and *dotted lines* indicate poor electron transfer. Cox, cytochrome c oxidase; Cyd, *bd* quinol oxidases; Cyt *c*_6_, cytochrome *c*_6_; PC, plastocyanin; PQ, plastoquinone; PQH_2_, plastoquinol; OEC, oxygen evolving complex; PSII, photosystem II

In the PSI-less/ScpABCDE^−^ mutant, the photon flux density exceeds the photosynthetic capacity even at low light. In the absence of the SCPs, chlorophyll-binding proteins are destabilized (Xu et al. 2002, 2004; Hernandez-Prieto et al. 2011) leading to photoinhibition of PSII and ^1^O_2_ production (Fig. 6a). PSII-generated ^1^O_2_ induces the loss of PSII activity, bleaching, and decreased growth and fitness observed in the PSI-less/ScpABCDE^−^ strain (Tibiletti et al. 2016). In the presence of NaCl, however, the electrons generated by PSII in the PSI-less/ScpABCDE^−^ mutant are efficiently absorbed and consumed by Cox and Cyd, as well as by other electron acceptors (Fig. 6b). In the PSI-less/ScpABCDE^−^ mutant, PSII electron sinks generate a shield against ^1^O_2_ and avoid cellular damages. A decreased amount of ^1^O_2_ in the presence of NaCl seems to enhance cell energization, the C catabolism as well as the N uptake, increased protein synthesis, and Chl biosynthesis leading to higher PSII assembly.

## Conclusions

SCPs, by stabilizing chlorophyll-binding proteins and PSII assembly, protect PSII from photoinhibitory damages. In the absence of SCPs, electrons accumulate and will lead to ROS formation. Here we show that the presence of 0.2 M NaCl induces increased respiratory activity (salt respiration) in the PSI-less/ScpABCDE^−^ that consumes the excess of PSII-generated electrons, thereby decreasing the electron flow through the electron transport chain, reducing the occurrence of charge recombination, and preventing ^1^O_2_ production.

## Electronic supplementary material

Below is the link to the electronic supplementary material.

**Supplementary Fig. 1** Color appearance of the PSI-less and PSI-less/ScpABCDE^−^ mutants grown in BG-11 supplemented with 10 mM glucose at either pH 8 (standard conditions), pH 6.5 or pH 9 (upper panel) and at pH 8 in the absence or presence of 0.5 mM NaHCO_3_ (lower panel) (PDF 134 KB)

Supplementary material 2 (DOCX 13 KB)
