# Supplementary figures and images for: The stress-induced SCP/HLIP family of small light-harvesting-like proteins (ScpABCDE) protects Photosystem II from photoinhibitory damages in the cyanobacterium Synechocystis sp. PCC 6803
Source: Photosynth Res. 2017 Aug 9;135(1):103–14. doi: 10.1007/s11120-017-0426-3 (PMC5783992; doi:10.1007/s11120-017-0426-3)

Supplementary  
Figure 1

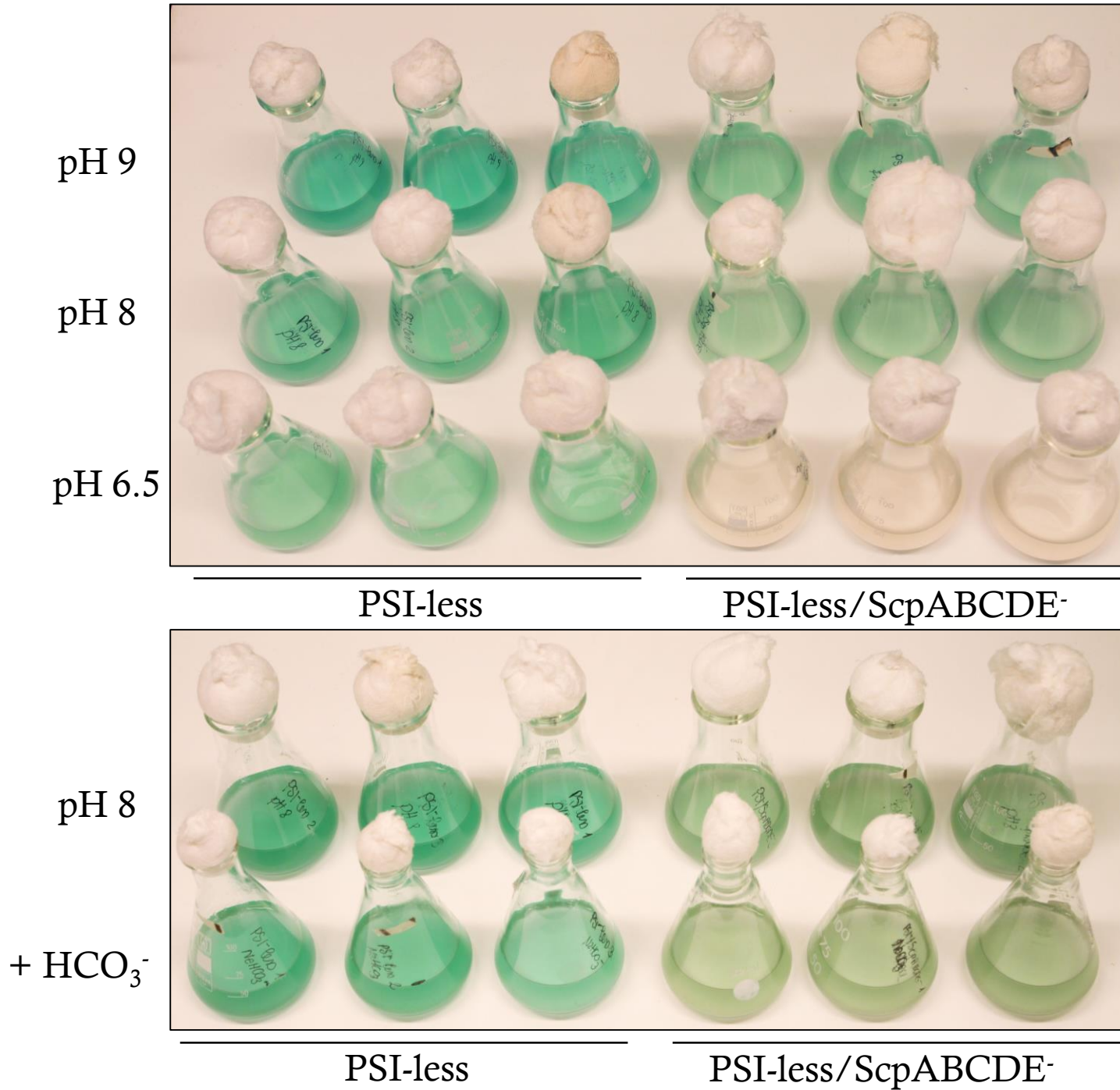

Supplement: Supplementary file 1 — Supplementary Fig. 1 Color appearance of the PSI-less and PSI-less/ScpABCDE− mutants grown in BG-11 supplemented with 10 mM glucose at either pH 8 (standard conditions), pH 6.5 or pH 9 (upper panel) and at pH 8 in the absence or presence of 0.5 mM NaHCO3 (lower panel) (PDF 134 KB) [file 11120_2017_426_MOESM1_ESM.pdf]
