# Supplementary material for: The stress-induced SCP/HLIP family of small light-harvesting-like proteins (ScpABCDE) protects Photosystem II from photoinhibitory damages in the cyanobacterium Synechocystis sp. PCC 6803
Source: Photosynth Res. 2017 Aug 9;135(1):103–14. doi: 10.1007/s11120-017-0426-3 (PMC5783992; doi:10.1007/s11120-017-0426-3)
Supplement: Supplementary file 2 — Supplementary material 2 (DOCX 13 KB) [file 11120_2017_426_MOESM2_ESM.docx]

**Supplementary Table 1.** Chlorophyll concentration per cell and relative doubling time of the PSI-less control strain and the PSI-less/ScpABCDE^-^ strain cultivated for 4 days at different pHs and in presence of 0.5 mM NaHCO_3_.

|  | **PSI-less** | | **PSI-less/ ScpABCDE^-^** | |
| --- | --- | --- | --- | --- |
|  | **fmol chl *a* / cell** | **division time (hrs)** | **fmol chl *a* / cell** | **division time (hrs)** |
| **pH 8** | 4.98 (±0.67) | 18.08 (±0.64) | 4.94 (±0.68) | 28.74 (±1.01) |
| **pH 6.5** | 4.68 (±0.38) | 19.50 (±1.26) | *n.d.* | *n.d.* |
| **pH 9** | 6.84 (±0.29) | 12.09 (±1.22) | 7.52 (±0.86) | 23.81 (±0.81) |
| **+ HCO_3_^-^** | 4.09 (±0.44) | 27.79 (±2.14) | 4.69 (±0.76) | 60.51 (±2.67) |

*Both strains were cultivated in BG11 supplemented with 10 mM glucose. Standard deviations with n ≥3 are given in brackets.
